# Anesthetic Management of a Pediatric Patient With Glucose-6-Phosphate Dehydrogenase Deficiency Undergoing Emergency Rigid Esophagoscopy: A Case Report

**DOI:** 10.7759/cureus.108677

**Published:** 2026-05-11

**Authors:** Ali Khaliq, Natasa Grancaric, Michael Girshin, Roni Mendonca, Benjamin J Suler

**Affiliations:** 1 Anesthesiology, New York Medical College, Metropolitan Hospital Center, New York, USA; 2 Anesthesiology, Oberlin College and Conservatory, Oberlin, USA

**Keywords:** esophageal foreign body, g6pd-deficiency, general anesthesia, methemoglobinemia, pediatric-anesthesia, rigid esophagoscopy

## Abstract

A 23-month-old, 8-kg boy with known glucose-6-phosphate dehydrogenase (G6PD) deficiency presented with a coin lodged in the upper esophagus and required urgent rigid esophagoscopy under general anesthesia. Anesthetic management was planned to avoid drugs with oxidative potential and to minimize physiologic stressors that could contribute to hemolysis or methemoglobinemia. General anesthesia was performed using agents considered safe in patients with G6PD deficiency, with intraoperative management focused on maintaining normoxia using the lowest possible inspired oxygen concentration needed to maintain oxygen saturation above 95%, normothermia, and hemodynamic stability. The foreign body was removed without complications, and there were no clinical signs of hemolysis or methemoglobinemia during hospitalization. Postoperative monitoring included serial hemoglobin assessment and clinical surveillance for jaundice and dark urine for more than 24 hours, with no evidence of delayed hemolysis. A follow-up phone call at 72 hours confirmed that there were no symptoms. This case highlights a practical perioperative approach to emergent pediatric anesthesia in a patient with G6PD deficiency, emphasizing careful drug selection and proactive prevention and monitoring of oxidative triggers.

## Introduction

Glucose-6-phosphate dehydrogenase (G6PD) deficiency is considered the most prevalent red cell enzymopathy globally and increases vulnerability to oxidative hemolysis when affected individuals are exposed to triggers such as infection, specific drugs, and physiologic or psychological stress [1,2]. G6PD is the rate-limiting enzyme of the pentose phosphate pathway, which generates nicotinamide adenine dinucleotide phosphate (NADPH). NADPH is required to maintain glutathione in its reduced form, allowing red blood cells to neutralize oxidative stress. Because mature erythrocytes lack mitochondria, they depend heavily on this pathway for antioxidant defense. In G6PD deficiency, reduced NADPH production limits glutathione regeneration, making erythrocytes vulnerable to oxidative injury, Heinz body formation, membrane damage, and hemolysis [2]. In the perioperative setting, risk arises from both oxidant or methemoglobinemia-associated medications and physiological factors such as hypoxia, acidosis, hyperthermia, and infection [2,3].

Although multiple reports describe anesthetic drug choices in G6PD deficiency, emergent pediatric airway-adjacent procedures, such as rigid esophagoscopy, have a distinct risk profile because fasting, aspiration risk, and time pressure can increase exposure to physiologic stressors [4]. While case reports exist for pediatric G6PD management in elective or non-emergent surgeries such as pyeloplasty and adenoidectomy, reports on emergency foreign body removal via rigid esophagoscopy in toddlers are scarce. This report contributes a practical, stepwise perioperative framework for emergency pediatric anesthesia in G6PD deficiency that integrates drug selection, avoidance of methemoglobinemia-associated and physiological triggers, and a monitoring plan for delayed hemolysis after discharge, addressing a gap in the literature for such high-acuity scenarios.

## Case presentation

**Figure 1:**
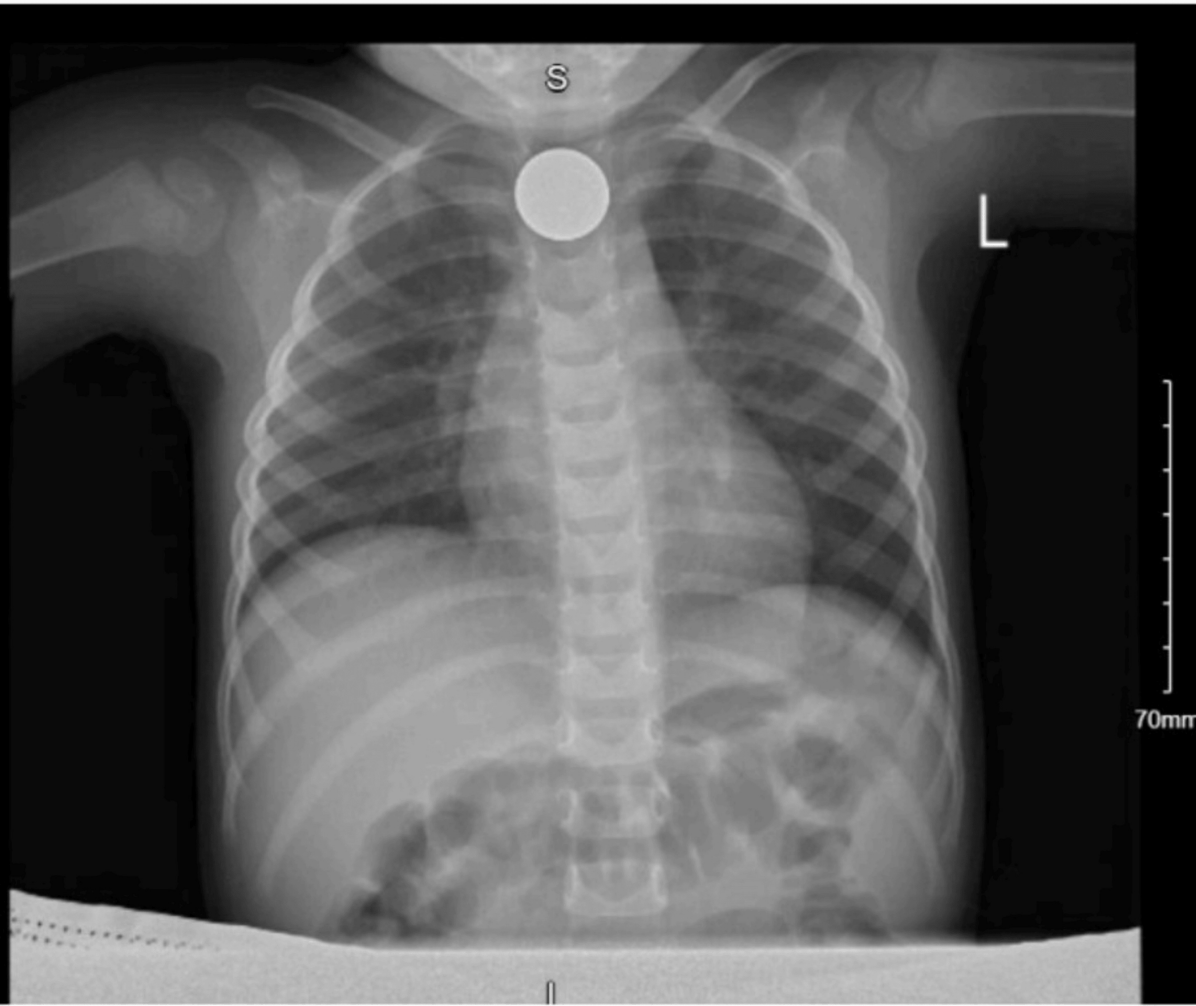
Preoperative chest X-ray demonstrating a round radiopaque foreign body, consistent with a coin, lodged in the upper esophagus.

A 23-month-old male child, weighing 8 kg, presented to the ED after swallowing a coin. Radiographic imaging confirmed the presence of a foreign body in the upper esophagus (Figure 1). The child was scheduled for urgent rigid esophagoscopy under general anesthesia.

Past medical history was significant for G6PD deficiency diagnosed in infancy. The most recent documented quantitative G6PD enzyme activity was 3.5 U/g Hb and was consistent with moderate deficiency (WHO Class III) [4]. The child had no prior documented hemolytic crises. Ethnicity was not available in the medical record at the time of emergency presentation. There was no family history of anesthetic complications. Baseline evaluation showed hemoglobin of 11.4 g/dL, with no clinical evidence of active hemolysis. The patient was afebrile at presentation, with a recorded temperature of 37.1°C. Because hemolysis can be delayed in G6PD deficiency [2], markers relevant to hemolysis were reviewed and obtained, including bilirubin of 0.5 mg/dL and lactate dehydrogenase (LDH) of 200 U/L. Urine appearance and urinalysis were negative for hemoglobinuria.

Perioperative differential considerations included aspiration risk due to the esophageal foreign body and uncertain fasting status, as well as the potential for hypoxia, acidosis, and hyperthermia, which can precipitate oxidative hemolysis in the setting of G6PD deficiency. Perioperative risk stratification was performed based on the confirmed baseline moderate G6PD deficiency.

After applying American Society of Anesthesiologists (ASA) standard monitors, including EKG, non-invasive blood pressure, pulse oximetry, and axillary temperature monitoring, inhalational general anesthesia was induced with sevoflurane in an oxygen/air mixture via facemask while maintaining spontaneous ventilation until IV access was established. After IV placement, anesthesia was deepened with fentanyl (2 mcg/kg; total, 16 mcg) and propofol (3 mg/kg; total, 24 mg), and neuromuscular blockade was achieved with rocuronium (0.6 mg/kg; total, 4.8 mg) to facilitate endotracheal intubation. Anesthesia was maintained with sevoflurane at 2%-3% (minimum alveolar concentration (MAC), 0.9-1.3) in 40%-60% oxygen in air.

Ventilation was managed to avoid physiological triggers associated with oxidative stress by maintaining normoxia and normocapnia and avoiding prolonged hyperoxia. FiO₂ was titrated to 30%-40% to maintain oxygen saturation >95%. End-tidal CO₂ was maintained between 35 and 40 mmHg, and temperature was actively managed to maintain normothermia using a forced-air warming system.

**Figure 2:**
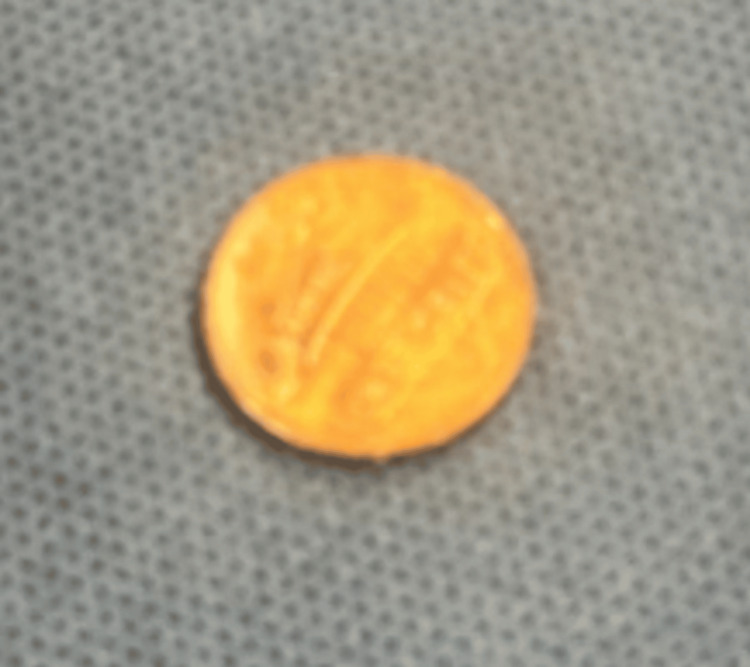
Extracted foreign body, consistent with a penny, recovered during rigid esophagoscopy, confirming complete removal from the upper esophagus without procedural complications.

The procedure was completed in approximately 35 minutes without complications, and the foreign body was successfully extracted by the esophagoscopist (Figure 2). The patient remained hemodynamically stable. No intraoperative evidence suggested hemolysis or methemoglobinemia, as determined by continuous clinical monitoring, including no unexplained desaturation, dark urine, or hemodynamic instability. No urinary catheter was placed because of the short procedure duration and low risk; urine color was assessed after extubation.

**Figure 3:**
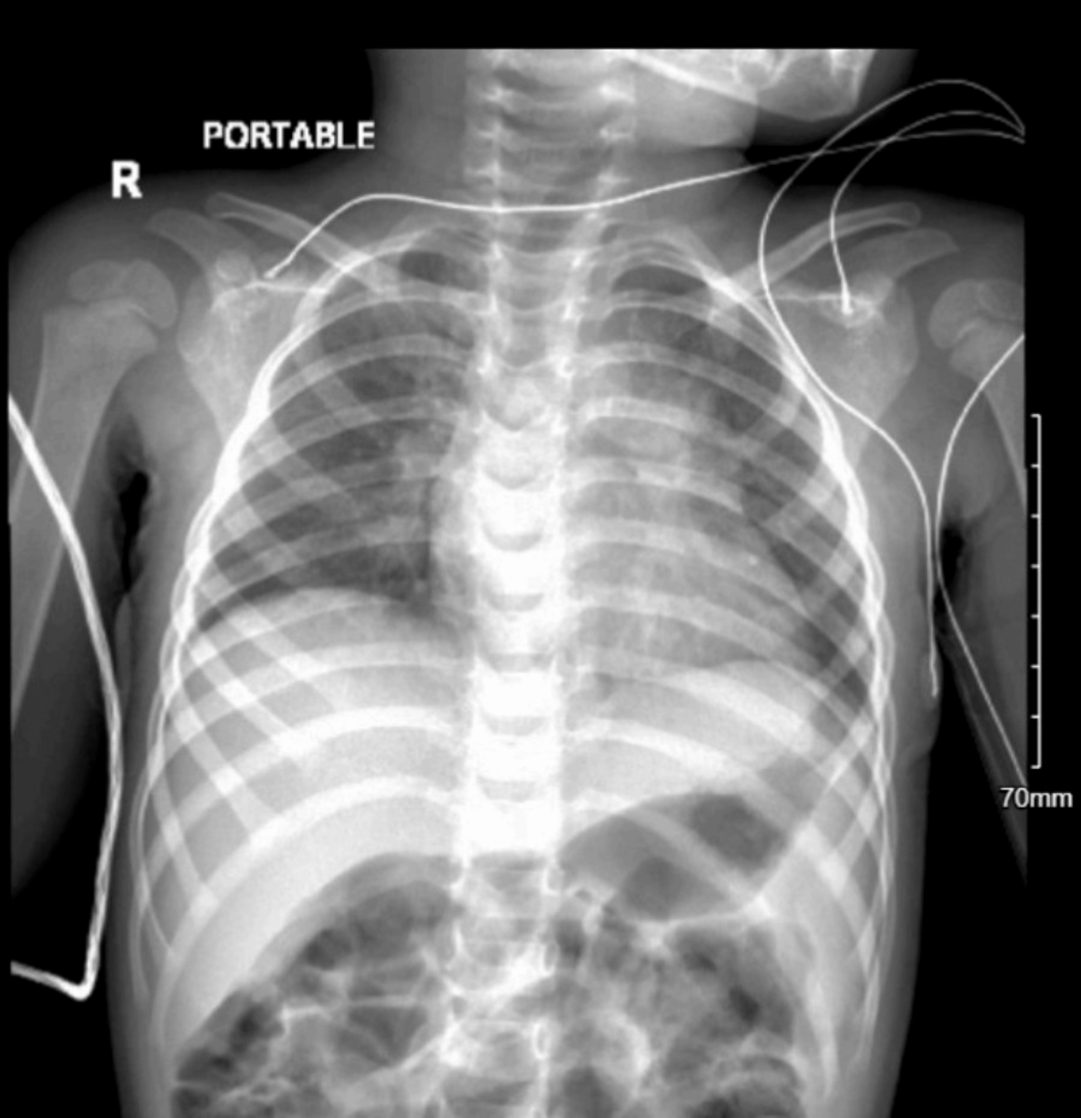
Postoperative chest X-ray showing complete removal of the foreign body with clear esophageal passage and no residual radiopaque density, verifying successful intervention.

Extubation was successful, and the child was transferred to recovery with close monitoring. Postoperatively, hemoglobin remained stable on serial checks: 11.3 g/dL at 6 hours and 11.4 g/dL at 24 hours. The child had no clinical signs of hemolysis, such as jaundice, dark urine, or tachycardia. Post-procedure radiography confirmed removal of the foreign body (Figure 3). Caregivers were instructed to monitor for delayed hemolysis, including jaundice, dark urine, lethargy, or poor feeding, for 72 hours after discharge. A follow-up phone call at 72 hours confirmed no delayed symptoms, a return to baseline activity and appetite, and no further hemolytic events.

## Discussion

This case illustrates emergency pediatric anesthetic management for rigid esophagoscopy in a child with G6PD deficiency, where risk is driven not only by drug selection but also by physiologic stressors. Because G6PD deficiency reduces NADPH production and impairs glutathione regeneration, patients are more likely to experience oxidative damage; perioperative care must therefore reduce oxidant exposures and avoid hypoxia, acidosis, and hyperthermia [1,2].

Physiologic stress response as an oxidative trigger

Emergency procedures may amplify catecholamine and inflammatory stress responses and increase the likelihood of transient hypoxemia or temperature dysregulation, particularly during transfer to a colder operating room environment and the administration of general anesthesia. Operative stress, infection, and metabolic disturbances, including acidosis, are recognized triggers for oxidative injury in patients with G6PD deficiency [2,3,4]. In this patient, oxygenation, ventilation, and temperature were all closely controlled, with SpO₂ kept at 98%-100%, EtCO₂ at 35-40 mmHg, and temperature at 36.5°C-37°C to limit the risk of oxidative stress while preserving procedural safety [2,3]. The emergent rigid esophagoscopy context, with aspiration risk, uncertain fasting status, and the need for rapid airway control, heightens the importance of avoiding desaturation and hemodynamic instability. This analytical approach reconciles the emergent context with G6PD-related risks, differing from elective cases where stressors are more controllable.

Drug selection and in vitro enzyme inhibition

Although some anesthetic-related agents inhibit G6PD activity in vitro, short perioperative use at typical clinical doses has not reliably resulted in hemolysis in published reports, and evidence-based reviews indicate that only a small group of medications are clearly contraindicated [3]. Accordingly, drug selection should prioritize clinical outcomes and established guidance, while recognizing that risk differs across G6PD variants and baseline enzyme activity levels [1,2,3]. Here, sevoflurane, propofol, fentanyl, and rocuronium were chosen because prior perioperative case reports describe their use without hemolytic complications in G6PD-deficient patients [4,5]. This case critically appraises the evidence, noting that in vitro data often overestimate risk compared with real-world outcomes.

Benzodiazepines, sevoflurane, and stress

Reports citing potential benzodiazepine-related G6PD inhibition are based primarily on in vitro enzyme-inhibition findings rather than consistent clinical hemolysis outcomes [5,6]. In this case, benzodiazepines were not required because the patient was not anxious. When anxiolysis is needed, the theoretical enzyme-inhibition risk should be weighed against the established risk that unmanaged stress, crying, and hypoventilation can lead to hypoxia and metabolic disturbances that may independently trigger hemolysis [2,4,5]. Sevoflurane was chosen for its favorable profile in G6PD deficiency, supported by clinical data over in vitro concerns. This highlights the importance of individualized risk-benefit assessment rather than a purely exclusion-based approach to drug selection.

Methemoglobinemia and topical agents

Topical benzocaine and prilocaine preparations are well-established causes of acquired methemoglobinemia [7,8]. Methemoglobinemia is particularly concerning in G6PD deficiency because methylene blue, the usual antidote, depends on NADPH generated via the G6PD pathway [8]. We therefore specifically avoided benzocaine- or prilocaine-containing topical anesthetics when alternatives, such as placement of an IV line after induction, were available and did not administer methylene blue, which can paradoxically worsen hemolysis in G6PD-deficient patients. If topical anesthesia is necessary, such as to facilitate IV placement, agents that do not contain prilocaine are preferred [7,9]. Published reviews have identified several medications and oxidant exposures that should be avoided or used with caution in patients with G6PD deficiency (Table 1) [3,10].

**Table 1:** Examples of medications to avoid or use with caution in G6PD deficiency (variant-dependent risk). Table created by the authors using data from Youngster I et al. [3] and Haka D et al. [10]. G6PD: Glucose-6-phosphate dehydrogenase.

Category	Medication / Class	Primary concern / Note
Higher risk	Dapsone	Oxidative hemolysis risk
	Primaquine	
	Nitrofurantoin	
	Sulfonamide antibiotics	
	Nalidixic acid	
	Methylene blue	Can worsen hemolysis in G6PD deficiency; also problematic if used for methemoglobinemia
	Topical anesthetics linked to methemoglobinemia, such as benzocaine or prilocaine	Methemoglobinemia risk; avoid in G6PD deficiency when alternatives exist
Note	Variant-, dose-, and context-dependent	Risk depends on variant class, dose, and clinical context; list is not exhaustive

Monitoring and delayed hemolysis

Hemolysis may occur hours after an oxidative exposure, with clinically detectable hemolysis often emerging within 24 to 72 hours and, in some contexts, worsening over subsequent days [9,11]. Postoperative surveillance should therefore extend beyond the operating room. In this case, we monitored for delayed hemolysis with serial hemoglobin measurements at 6 and 24 hours and clinical assessment for jaundice and dark urine during a 24-hour observation period. Caregivers were instructed to watch for jaundice, dark urine, or lethargy for 72 hours after discharge, with follow-up confirming the absence of delayed symptoms [2,9,11].

Practical take-home framework for emergency pediatric anesthesia in G6PD deficiency

Based on this case and the existing literature, a practical perioperative framework for similar emergencies includes confirming baseline status when feasible by documenting hemoglobin, hemolysis markers, including bilirubin, LDH, and urinalysis, and enzyme activity or class, if known [2,3]. Agents with reassuring clinical safety data should be selected, while oxidant and methemoglobinemia-associated drugs should be avoided when alternatives exist [2,3-6]. Physiologic triggers should be prevented by maintaining normoxia with titrated FiO₂, normocapnia, and normothermia, and by aggressively treating infection or fever [2,4,5]. Delayed hemolysis monitoring should also be planned with serial hemoglobin checks when appropriate, along with explicit discharge instructions for 72-hour symptom surveillance [2,12,13].

Limitations

This is a single case report, and broader conclusions about comparative drug safety in G6PD-deficient patients cannot be drawn. Quantitative enzyme activity and variant class were partially characterized, but genetic data were incomplete, and follow-up beyond 72 hours was limited. This case illustrates how current evidence on G6PD-deficient patients can be applied to emergency-focused perioperative plans that emphasize both appropriate medication selection and minimization of physiologic stressors [2-4].

## Conclusions

Children with G6PD deficiency can undergo emergency rigid esophagoscopy under general anesthesia safely when management focuses on meticulous preparation, avoidance of oxidant and methemoglobinemia-associated drugs, and vigilant perioperative prevention and monitoring. This case contributes to the literature by providing a practical framework for perioperative decision-making in time-sensitive pediatric airway-adjacent procedures in the setting of G6PD deficiency.
